# Hyperbaric Oxygen Therapy in Branch Retinal Artery Occlusion in a 15-Year-Old Boy with Methylenetetrahydrofolate Reductase Mutation

**DOI:** 10.1155/2015/640247

**Published:** 2015-02-05

**Authors:** Ali Riza Cenk Celebi, Sibel Kadayifcilar, Bora Eldem

**Affiliations:** ^1^Department of Ophthalmology, Acibadem University School of Medicine, 34303 Istanbul, Turkey; ^2^Department of Ophthalmology, Hacettepe University School of Medicine, Ankara, Turkey

## Abstract

*Purpose.* To report the efficacy of hyperbaric oxygen (HBO) therapy in a case of branch retinal artery occlusion (BRAO) in a 15-year-old boy. *Methods.* We report a 15-year-old boy with sudden loss of vision due to BRAO. Examination included laboratory evaluation for systemic risk factors. Follow-up exams included visual acuity, fundus examination, fundus fluorescein angiography, and visual field testing. HBO therapy was employed for treatment. *Results.* Medical history was positive for isolated glucocorticoid deficiency. Laboratory evaluation disclosed hyperhomocysteinemia and methylenetetrahydrofolate reductase (MTHFR) mutation. The visual acuity 0.05 at presentation improved to 0.8 after 20 days of HBO therapy. There was no change on visual fields. *Conclusion.* In this pediatric case, HBO therapy was useful in the treatment of BRAO.

## 1. Introduction

Retinal artery occlusion (RAO) is a common vision-threatening disease affecting primarily patients older than 60 years. Embolism, atheroslerotic plaque, and intraluminal thrombosis have been suggested to play a role in the pathophysiology of RAO [1]. Increased serum homocysteine has been indicated as a risk factor in retinal vascular occlusive disease [1]. Weger and associates found the relationship between hyperhomocysteinemia and MTHFR mutation in retinal artery occlusion [2]. A number of therapies had been used in the treatment of retinal arterial occlusive diseases. These included carbogen inhalation, acetazolamide infusion, ocular massage, and paracentesis, as well as vasodilators such as intravenous glyceryl trinitrate [3]. None of these have been shown to alter the natural history of disease definitively. In retinal arterial occlusions either branch or central, vision loss results from cell death in the inner retinal layers despite relative sparing of the outer layers. If supplemental oxygen is provided, however, oxygen from the choroidal circulation may diffuse in adequate quantity to the inner layers of the retina to maintain retinal function and restore vision [4]. Hyperbaric oxygen (HBO) therapy uses intermittent breathing of 100% oxygen at pressures >1 atmosphere absolute (ATA). The therapeutic effect of HBO is attributable to the mechanical effect of increased environmental pressure on gas-containing spaces in the body and the physiologic changes induced by hyperoxia. The inspiration of high levels of oxygen has a negligible impact on the total hemoglobin oxygen content. However, HBO increases the amount of oxygen dissolved in plasma, from 0.32 to 6 mL O_2_/100 mL of blood when breathing 100% O_2_ at 3 ATA. This considerable increase in the amount of oxygen made available to the tissues is of great importance when tissue oxygenation is impaired [4]. The emergent treatment of occlusive vascular disease of the retina nowadays is HBO therapy given early for eligible patients.

We hereby report branch retinal artery occlusion (BRAO) associated with MTHFR mutation in a 15-year-old boy treated with HBO.

## 2. Case Presentation

**Figure 1:**
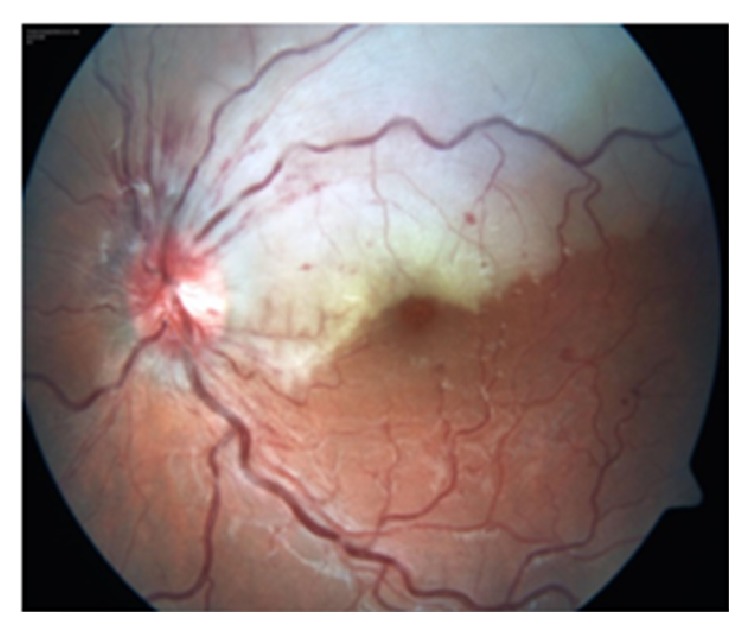
Retinal edema with occasional hemorrhages in the upper temporal quadrant of the left eye.

**Figure 2:**
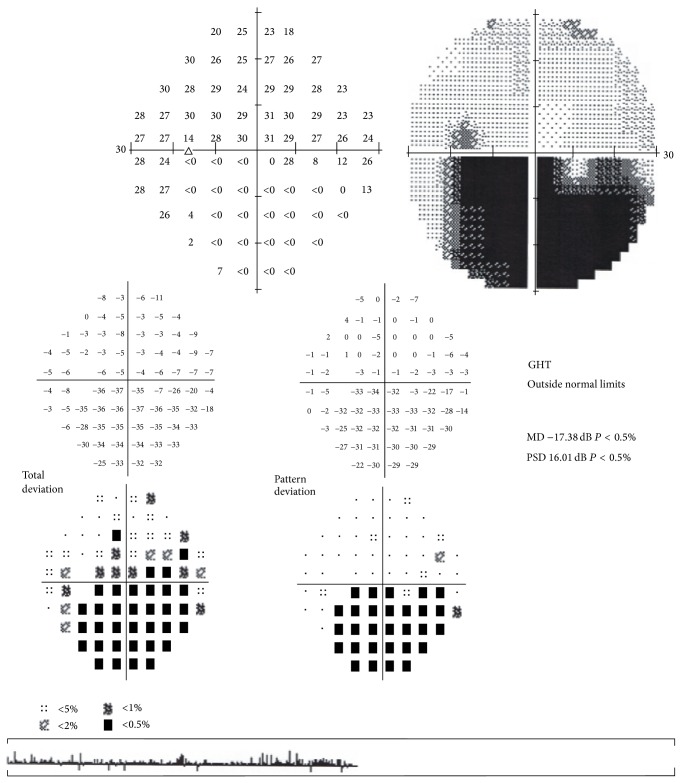
Corresponding visual field defect in the lower quadrant.

**Figure 3:**
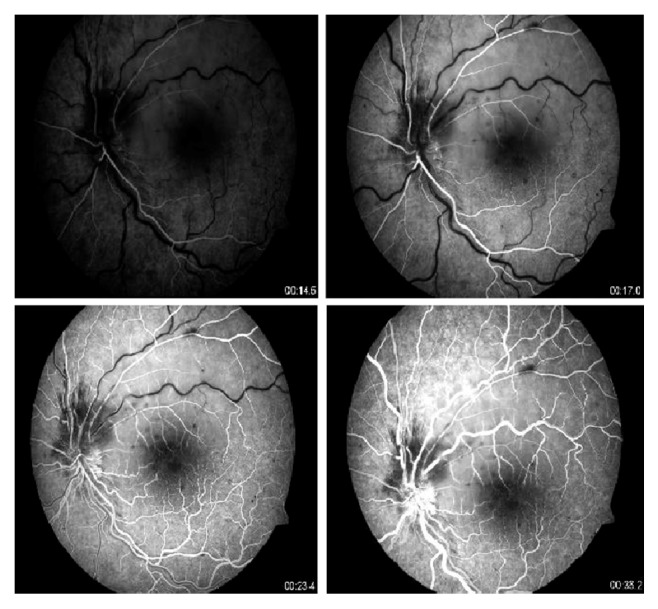
Delayed filling of the affected artery and hypofluorescence in the surrounding retina on FFA. Numbers indicate seconds.

**Figure 4:**
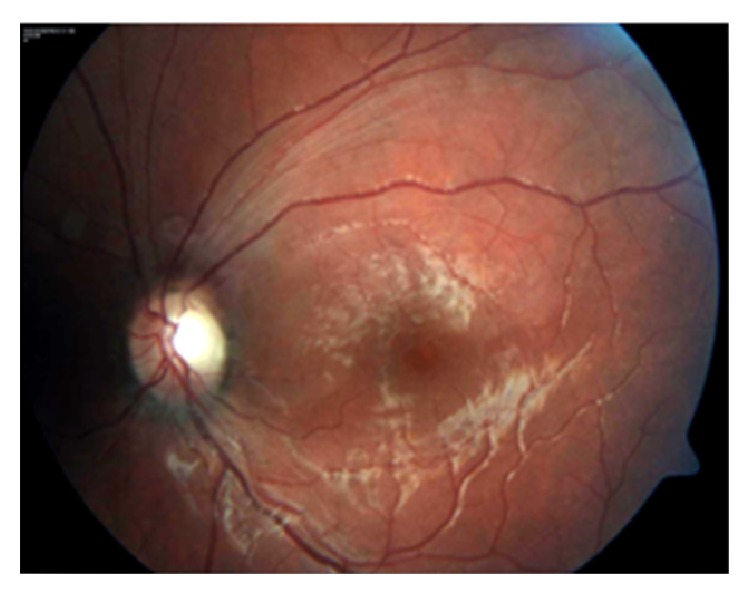
Improvement of retinal edema 20 days later.

**Figure 5:**
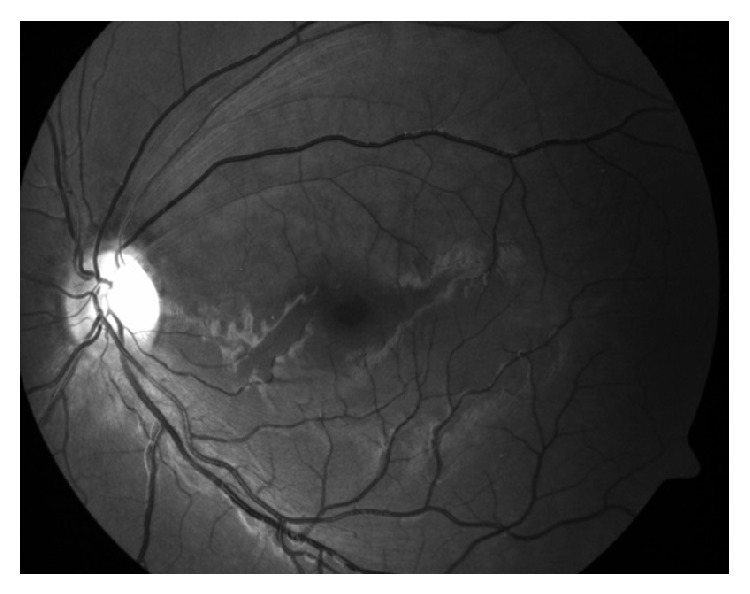
Red free image disclosing retinal nerve fiber loss in the affected area 6 months later.

A 15-year-old boy was brought by his parents to Hacettepe University Children's Hospital Emergency Room on a Friday night for painless sudden visual loss in the left eye for the last 12 hours. The full ophthalmologic and systemic examination was followed by laboratory evaluation for systemic risk factors. Follow-up exams included visual acuity, fundus examination, visual fields, fundus fluorescein angiography (FFA) on following Monday, and additional laboratory testing. The vision in the left eye was 0.05 and 1.0 in the right on admission. Slit lamp examination of both eyes and dilated fundus exam of the right eye were within normal limits. Dilated fundus examination of the left eye showed retinal edema in the upper quadrant suggesting an upper temporal retinal branch artery occlusion (Figure 1). The confrontation visual field examination disclosed lower hemifield defect that was confirmed the following day with Humphrey perimeter (Figure 2). FFA showed delayed filling of the affected artery and hypofluorescence in the surrounding retina (Figure 3). Past medical history revealed isolated glucocorticoid deficiency diagnosed 8 years ago. The family history was negative for coagulopathies. His preliminary laboratory testing including hemogram, biochemistry, and erythrocyte sedimentation rate was within normal limits. The patient was given HBO at that night and for 20 days more. The following day, the visual acuity of the left eye improved to 0.4. Systemic examination including echocardiography was within normal limits. Further laboratory evaluation disclosed increased homocysteine levels of 20.9 micromol/L (normal range: 5.5–17). Genetic testing revealed homozygous MTHFR mutation (677 C→T). Patient's visual acuity in the left eye after 20 sessions of HBO therapy was 0.8, dilated fundus examination findings on left eye showed marked improvement of the retinal edema (Figure 4), but visual fields remained the same. 300 mg/day acetyl salicylic acid (ASA) and 1 mg/day folic acid were recommended by the Pediatric Hematology Department. As a result of daily folate supplements, patient's plasma homocystein levels decreased to 8.51 micromol/L (normal range: 5.5–17). Final examination 6 months later disclosed loss of retinal nerve fibers in the affected area though the visual acuity was still 0.8 (Figure 5).

## 3. Discussion

RAO is mostly seen in the elderly with clinical findings suggestive of atheromatous emboli. It is uncommon in the young population. Information regarding risk factors in this age group is scant. Multifactorial etiology included cardiac, valvular, and vascular inflammatory disorders [3]. Wenzler and associates suggested that hyperhomocysteinemia is a risk factor for retinal vascular occlusive disease [1]. It was the most common cause of RAO in young individuals in India [5].

Hyperhomocysteinemia results from various disorders including cystathionine b-synthase deficiency, MTHFR mutation, and defects in the metabolism of folate and vitamin B12. As a cause of RAO in children, the genetic predisposition to thrombosis should be investigated. MTHFR gene polymorphism reduces MTHFR enzyme activity and may cause hyperhomocysteinemia, which affects the vascular endothelium, and may induce occlusive vascular disease [2]. Homozygosity for the MTHFR C677→T mutation has been suggested to cause hyperhomocysteinemia [6]. Reducing plasma homocysteine levels by 25% is easily achieved by a low dose of folic acid [7]. Thus, treatment of hyperhomocysteinemia with folic acid is commonly employed.

Various treatment modalities have been tried in the treatment of RAO. Supplemental oxygen showed promising visual results. If supplemental oxygen is provided in RAO, oxygen from the choroidal circulation may diffuse in adequate quantity to the inner layers of the retina to maintain retinal function and restore vision. The challenge is to provide the supplemental oxygen early enough after the onset of vision loss to prevent irreversible damage to the retina. In experimental models of complete central RAO, the ischemic time window before permanent retinal damage occurs is just over 90 minutes; in the clinical setting where occlusion may be incomplete, return of vision may be achieved even after delays of eight to 24 hours [4]. In patients with RAO presenting within 24 hours of vision loss, supplemental oxygen should be started immediately. If the patient responds to HBO, follow-up treatment with supplemental oxygen should be customized to maintain retinal viability until the obstructed retinal artery recanalizes, which typically occurs within the first 72 hours. Currently, eye diseases are among the off-label uses of HBO. However, there is increasing evidence showing its safety and efficacy in RAO, cystoid macular edema secondary to retinal vein occlusion, scleral thinning and necrosis faced after pterygium surgery, orbital rhinocerebral mucormycosis, nonhealing corneal edema, and anterior segment ischemia in ophthalmology [8]. Visual function should be monitored as indicated before, during, and after HBO therapy. Major parameters for visual prognosis are the time lag from the onset of symptoms to the beginning of HBO treatment and the time lag until retinal reperfusion begins. Waisman and colleagues described the use and safety of HBO treatment in children between 2 months and 18 years of age [9]. None of these 139 children had an ophthalmic indication. To the best of our knowledge ours is the first case of HBO treatment for branch retinal artery occlusion in a child with known MTHFR mutation.

In order to define the role of HBO therapy in RAO further studies in large case groups are necessary.
